# Positive impact of sleep on recall of multiplication facts

**DOI:** 10.1098/rsos.230663

**Published:** 2023-09-27

**Authors:** Jayne Spiller, Camilla Gilmore

**Affiliations:** ^1^ School of Psychology, University of Leicester, Leicester LE1 7RH, UK; ^2^ Centre for Mathematical Cognition, University of Loughborough, Loughborough, UK

**Keywords:** sleep, learning, multiplication, mathematics

## Abstract

This study tested the hypothesis that learning complex multiplication problems (e.g. 8 × 23 = 184) prior to sleep would benefit recall in adult participants compared with learning the problems prior to a period of wakefulness. This study used a within-participant design where all participants learnt complex multiplication problems in two conditions separated by one week. In one condition, learning was before bed (sleep learning condition) and in the other condition learning was in the morning (wake learning condition). In each condition, recall was tested approximately 10.5 h later. Data were collected online from 77 participants. In the subset of the sample with greater than or equal to 60% accuracy at the initial learning session (*n* = 37), the sleep learning condition participants had better recall compared with the wake learning condition. This equated to a moderate effect size, Cohen's *d* = 0.51. Regardless of initial levels of learning (*n* = 70) the same beneficial effect of sleep on recall was found with a small effect size, Cohen's *d* = 0.33. This study has identified a beneficial effect of learning prior to sleep on recall of complex multiplication problems compared with learning these problems during the daytime. Future research should explore whether similar effects are observed with children learning simple multiplication facts.

## 1. Introduction

The beneficial effects of sleep on memory are well established. Sleep supports the consolidation of learnt facts, shown by improved recall of word pairs by both children and adults when learning occurred prior to sleep, compared with learning followed by a period of daytime waking [1,2]. There is a moderate beneficial effect size for recalling novel word pairs, where the second word in a pair is recalled when the first word of the pair is presented following sleep versus wake (Hedges *g* = 0.57) [3]. The vast majority of research investigating the impact of sleep on memory processes has been focused on word learning, which draws on declarative memory. However, sleep has also been shown to benefit spatial cognition [4] and procedural memory in young adults, such as memorization of a spatial sequence [5]. However, there are many other domains in which memory processes play an important role, for example, in mathematics.

It is recognized that learning and performing mathematics draws heavily on memory. For example, there is a close relationship between mathematical achievement and both short-term and working memory [6]. On the basis of this association, educational researchers have a growing interest in how memory management techniques can be applied within the classroom [7]. While memory processes are implicated in a wide range of mathematical skills [8], one evident role concerns the memorization and retrieval of number facts (e.g. number bonds, multiplication tables). Being able to correctly recall number facts from memory is an important component of mathematical achievement [9]. Fluent retrieval of number facts frees up cognitive resources that would otherwise be required to derive the answers to simple calculations, allowing individuals to focus on conceptual and strategic aspects of maths. This is recognized by the UK government which has recently introduced a new statutory national multiplication tables test to be taken by all children aged 8–9 years old. Given the critical role of memory in mathematics education, it is valuable to identify factors that can support learning and retrieval. No studies have yet explored whether the learning and recall of number facts, such as multiplication tables, are improved following sleep. This research is therefore timely.

While it might be plausible that the well-established beneficial effects of sleep on word learning could directly extend to learning number facts, there are some important differences between the domains. Multiplication fact learning is not a purely declarative process, as strategies such as decomposition, a standard algorithm or repeated addition can be used to solve complex multiplication problems [10]. However, the fact that the same strategies from simple multiplications can be used to solve complex multiplications demonstrates that learning complex multiplications is not without a familiar context. Learning stimuli that adhere to a familiar structure may particularly benefit from sleep-related memory consolidation. Indeed in the word-learning literature, it has been demonstrated that using nonsense words that follow familiar lexical patterns shows a greater sleep-related recall benefit (moderate effect size) than words in a foreign language that do not follow familiar patterns (small effect size) [3]. Where participants are given the opportunity to engage with the complex multiplication stimuli using familiar strategies, through the use of untimed trials in the first instance, it may enhance sleep-related benefit compared with no opportunity to consider the complex multiplication stimuli as a continuum of simple multiplication, in which only timed trials are used to constrain the strategy to rote memorization.

As discussed, initial engagement with complex multiplication stimuli is likely to involve problem solving using strategies rather than through simple memorization. However, when the response time is constrained to ensure that participants use retrieval rather than strategies to associate the multiplication problem and its answer [11] multiplication facts can be learnt the same way that word-pairs are presented in other studies of sleep-related memory consolidation. Indeed, research suggests that rote memorization techniques are frequently used by children to learn multiplication problems as part of a range of strategies [12]. This raises the potential of leveraging sleep-associated benefits to improve children's multiplication fact knowledge.

Evidence from brain imaging studies supports the potential benefit to number fact learning from sleep due to the wide network of brain regions involved. Brain imaging following the drill learning of answers to complex multiplications indicates greater activation in the left angular gyrus for learnt answers versus novel problems [13–15]. There is brain imaging evidence to suggest that solving arithmetic problems using procedural knowledge, which relies on working memory versus fact retrieval from long-term memory recruits different brain areas [14]. Other evidence indicates the involvement of hippocampus and the para-hippocampal and retrosplenial structures in learnt problems compared with novel problems, with many of the frontal clusters activated but no significant difference in the activation of the angular gyrus between these two conditions [16]. The activation of a wide network of brain regions, including both the hippocampus and frontal cortex, suggests that the learning of arithmetic facts might benefit from sleep-related consolidation. The active consolidation system hypothesis, in which declarative memories in the hippocampus are reactivated and transferred to the neocortex, driven by slow wave sleep oscillations, would suggest that the transfer of arithmetic facts to long-term memory in the neocortex is optimized when learning is followed by a period of sleep, compared with a period of wakefulness.

In the current study, we investigated whether benefits of learning prior to sleep compared with learning prior to wakefulness were observed when adult participants learnt complex multiplication facts (i.e. with either multiplier or multiplicand > 12). Recall of the newly learned items was tested 10.5 h later. We predicted that there would be a significant interaction between condition (learning prior to sleep versus learning prior to wakefulness) and session (learning versus recall), specifically that there would be significantly higher accuracy in the recall session of the sleep learning condition compared with the wake learning condition.

Our learning sessions were designed following prior research of effective approaches. Evidence suggests that integrated training where both strategies are modelled and timed trials are used to teach the answers to simple multiplications for school children are effective in increasing and maintaining response accuracy [11]. Therefore, our learning sessions included blocks in which participants could use procedural strategies as well as those in which participants were required to retrieve the answer. Permitting participants to use untimed strategies such as decomposition, a standard algorithm or repeated addition (for definitions, see [10]) to solve the multiplication problem upon first presentation also reflects real-world learning, as less than 10% of participants used retrieval strategies on first presentation of complex multiplications [10]. We also employed an incremental rehearsal method (in which novel items are added one-by-one (see electronic supplementary material: https://osf.io/avy6d/files/osfstorage) which has shown a large effect size in the recall of simple multiplication problems relative to traditional drill, in which problems are repeated until all are correct [17].

## 2. Method

The design, sample size, exclusion criteria and analysis plan were pre-registered and can be found at https://aspredicted.org/3mb8d.pdf.

### 2.1. Participants

The participants were 184 adults who were resident in the UK and aged between 18 and 40 years. Recruitment strategies included targeting undergraduate students (excluding mathematics and physics students) via an online advert, recruitment via Prolific and psychology undergraduate students. Upon downloading the data, despite including a location check screening of the participants' IP addresses, 74 participants needed to be excluded due to having completed the experiment in a non-UK timezone. The UK residency requirement was to ensure that participants completed the learning and test sessions at set times in relation to their sleep schedule. Of the remaining 110 participants, *n* = 33 were excluded due to taking over 1 h 45 min to complete the learning session (*n* = 6), and/or completing the experiment at the incorrect time (*n* = 9), and/or participants reporting that their sleep on the night of the sleep learning condition was greater than 2 h shorter than usual (*n* = 20). These exclusion criteria were pre-registered.

A total of 77 participants were included in the final analysis. Participants received either monetary compensation for their time or course credit. This study was approved by the Loughborough University Ethics Review committee and all participants gave informed consent.

### 2.2. Design

The experiment had a within-participants design. All participants completed four experimental sessions: a learning session (scheduled between 20.00 and 22.45) followed by sleep, and a recall test, approximately 10.5 h later and a learning session (scheduled between 7.30 and 10.00) followed by a period of wakefulness then a recall test approximately 10.5 h later. Participants were randomly allocated to complete either the learning or sleep condition first and there was a gap of one week between the conditions.

### 2.3. Stimuli and procedure

Participants completed the experimental sessions online unsupervised using Gorilla. Participants were asked to complete the experiment in a quiet location where they would not be disturbed and to not listen to music while completing the experiment. Prior to completing the first learning session, participants answered 22 single digit multiplication problems (see electronic supplementary material for a list of these problems: https://osf.io/avy6d/files/osfstorage). Answers to the problems were considered correct if they were answered accurately within 6 s.

The complex multiplication facts used for the learning sessions included facts taken from the 22, 23, 24, 26, 27, 28, 29, 31, 32, 33 multiplication tables. The multiplier or the multiplicand was 6, 7, 8 or 9. There were no tie problems. Two problem sets were created. A pilot study (*n* = 14; between subjects) revealed no significant difference in learning between set A and set B. Therefore, the use of set A and B was counterbalanced across participants.

#### 2.3.1. Learning sessions

Participants completed nine blocks in which they learnt 14 new complex multiplication facts. In the first block of the learning session, seven complex multiplication facts were introduced. Each problem (e.g. 6 × 26 =) was first presented in an untimed trial and participants were asked to provide the answer. Feedback on accuracy was provided followed by the multiplication fact and correct answer being re-presented on screen (regardless of accuracy of participant's response). Following the untimed trial, the same problem was presented in a timed trial (time limit 6 s). Feedback indicated whether the answer was correct or not, the response was recorded as incorrect if the answer was false or recorded as timed out, and the problem and answer together were shown. Each of the seven new problems was shown first in an untimed and then timed format.

The second learning block included only timed trials. Feedback was provided and the problem was re-presented with the correct answer if incorrect/timed out. Once all seven problems were presented, any problems that were answered incorrectly on the first presentation were repeated. This process continued until all the problems had been answered correctly. The third block repeated this protocol.

In each of the six remaining learning blocks, one new multiplication problem was introduced. This was presented first in an untimed trial, followed by a timed trial. The repetition of the new problem was interspersed with seven facts learnt in the previous blocks, using the ratio of one new multiplication problem to seven known multiplication problems (i.e. previously answered correctly at least twice). This was repeated until all the new facts had been introduced (14 in total). See electronic supplementary material for a description of the incremental rehearsal procedure for these blocks on OSF: https://osf.io/avy6d/files/osfstorage. Each multiplication problem was presented at least 11 times across the learning blocks but the number of presentations will vary by participant according to their performance on the second and third learning blocks.

At the end of the learning session, there was a final test block. Participants completed one timed block of 28 multiplication problems (each of the 14 multiplication facts twice). Feedback was provided and the problem was re-presented with the correct answer if incorrect/timed out. Accuracy on this block was used as the learning session score. To reach criterion for inclusion in the analysis, participants needed to answer 60% of the 28 problems correctly within 6 s.

#### 2.3.2. Recall sessions

Approximately 10.5 h after each learning session participants completed one timed block of the 28 multiplication problems. Feedback was provided and the problem was re-presented with the correct answer if incorrect/timed out. Accuracy on this block was the recall session score.

At the beginning of each block, participants were reminded of the 6 s time limit and to not calculate the answers to the problems but to recall the answer from memory. They were informed that they needed to pay attention to the feedback given as they needed to remember the answers to the problems for the next set of trials. Following each learning session, participants also reported on their feelings of tiredness on a scale 1, not at all tired; 2, slightly tired; 3, moderately tired; 4, very tired; to 5, extremely tired. At the start of the sleep condition recall session, participants indicated the time that they went to bed, time they woke up and estimated time to fall asleep and whether their sleep duration was more than 2 h shorter than usual. At the end of the second recall session, they also stated the frequency with which they used non-retrieval strategies to answer the problems (never, the occasional problem, half of the time, most of the time, all of the time).

#### 2.3.3. Analysis

Forty participants achieved the pre-registered 60% criterion on the test block at the end of the first learning session (mean accuracy 78%). Outlier values for the difference between the learning and test sessions for each condition were identified as values 3rd quartile + 1.5 × interquartile range or 1st quartile – 1.5 × interquartile range. Three outlier cases were identified and excluded. Thus, the primary analysis was conducted on 37 participant cases. Sleep duration was calculated as the difference between bedtime and wake time minus time taken to fall asleep. Below we first report our pre-registered analyses followed by exploratory analyses. The ANOVAs were conducted using R version 4.1.3 using the rstatix package and the ggplot2 package was used to construct the graph. The remaining analyses were conducted in SPSS.

## 3. Results

Sixteen participants were assigned to the sleep condition first and 21 were assigned to the wake condition first. Learning scores did not differ between the sleep condition first and the wake condition first participants, all *p*'s > 0.06. There was also no significant difference in any of the learning or recall scores between participants who completed the learning session with set A first (*N* = 17) or set B first (*N* = 20), all *p*'s > 0.466.

### 3.1. Pre-registered analyses

**Figure 1.**
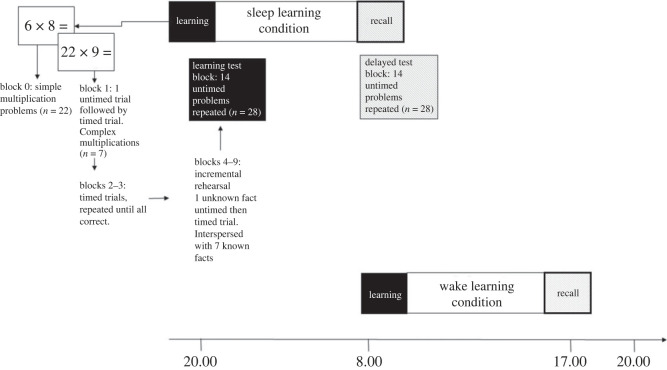
Overview of study design.

**Figure 2.**
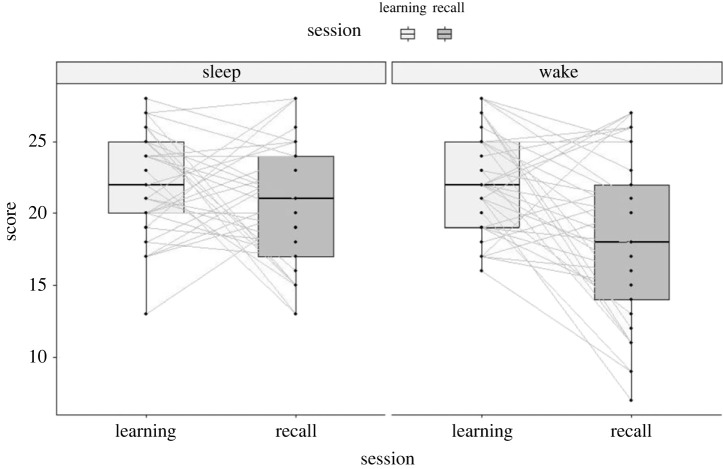
Boxplot and paired data points for scores on the learning and recall sessions for the learning prior to sleep and learning prior to wakefulness conditions.

A 2 (sleep, wake) × 2 (learning, recall) repeated measures ANOVA revealed significant effects of session (learning versus recall) *F*_1, 36_ = 21.90, *p* < 0.001 and condition (sleep versus wake) *F*_1,36_ = 4.34, *p* = 0.044 on accuracy. Importantly, there was also a significant interaction between session and condition *F*_1,36_ = 8.65, *p* = 0.006. *Post* *hoc* tests revealed significantly lower recall accuracy in the wake condition (M 18.46, s.d. 5.71) compared with the sleep condition (M 20.68, s.d. 4.22), *t* = 3.097, *p* = 0.004, significant after Bonferroni adjustment, Cohen's *d* = 0.51, moderate effect size [18] mean difference 2.22 (s.d. 4.35), equating to two additional facts recalled (see figures 1 and 2 and tables 1 and 2).

**Table 1.** Descriptive statistics for *N* = 37.

	sleep	wake	*p*-value
learning score (accuracy out of 28)	22.16 (3.56)	22.14 (3.55)	0.964
recall score (accuracy out of 28)	20.68 (4.22)	18.64 (5.71)	0.004
time between end of learning session and start of recall session (in minutes)	635.1 (61.74)	605 (69.88)	0.049
tiredness score after learning session (1 not at all tired to 5 extremely tired).	3.27 (1.12)	2.70 (1.08)	<0.001

**Table 2.** Proportions of participants who benefitted from the sleep learning condition above the wake learning condition.

	met criterion (*n* = 37) % (*n*)	did not meet criterion (*n* = 33) % (*n*)
better recall in wake condition	29.7 (11)	45.5 (15)
the same recall in sleep and wake conditions	10.8 (4)	9.1 (3)
better recall in sleep condition	59.5 (22)	45.5 (15)

The interval between the end of the learning session and start of the delayed test ranged from 485 to 750 min in the sleep condition and 410 to 728 min in the wake condition. A paired sample *T*-test revealed a significantly shorter interval in the wake condition, *t* = 2.04, *p* = 0.049, with a mean of 10 h 35 min for the sleep condition and 10 h and 5 min for the wake condition; however, both intervals were close to the 10 h 30 min interval which was pre-registered. Following the sleep learning session, participants reported significantly greater tiredness (modal response: moderately tired) compared with the wake learning session (modal response: slightly tired). However, tiredness ratings did not explain a significant proportion of variance in the sleep learning score: standardized *β* = −0.275, *t* = −1.70, *p* = 0.099, *R*^2^ = 0.076, nor the difference between the sleep learning score and the sleep recall score: standardized *β* = 0.098, *t* = 0.585, *p* = 0.562, *R*^2^ = 0.01.

Following the sleep learning session, participants' sleep was an average of 8 h and 0 min (s.d. 1 h 16 min). Sleep duration did not explain any significant difference between the sleep learning score and the sleep recall score: standardized *β* = −0.071, *t* = −0.420, *p* = 0.677, *R*^2^ = 0.005.

To establish whether participants followed the instructions to memorize the multiplication problems, at the end of the experiment they were asked whether they used non-retrieval strategies to provide the answer to the problem. The majority of participants never (32.4%) or occasionally (45.6%) used strategies to solve the problem, while 13.5% used strategies half of the time and 8.1% used strategies most of the time.

### 3.2. Exploratory analyses

#### 3.2.1. Is there a beneficial effect of learning followed by sleep for participants with lower levels of initial learning?

We pre-registered a criterion for inclusion that participants needed to achieve 60% accuracy on the test block at the end of the first learning session. In an exploratory analysis, we included all participants regardless of score on the first learning session. This increased the sample to 70, after excluding seven additional outlier cases. Mean accuracy on the first learning test for this wider sample was 60%. A repeated measures 2 × 2 ANOVA with the larger sample revealed a significant main effect of session *F*_1,69_ = 43.18, *p* < 0.001 but not of condition *F*_1,69_ = 0.65, *p* = 0.424. Importantly, there was a significant interaction between session and condition *F*_1,69_ = 11.33, *p* = 0.001. *Post* *hoc* test revealed significantly lower recall accuracy in the wake condition (M = 14.59, s.d. 6.51) compared with the sleep condition (M = 16.11, s.d. 6.51), for the recall scores *t* = 2.77, *p* = 0.007, significant after Bonferroni correction, Cohen's *d* = 0.33, small effect size [18], mean difference 1.53 (s.d. 4.62), equating to one additional fact recalled.

#### 3.2.2. Factors associated with learning scores and sleep benefits

Two measures give an indication of participants' prior mathematical proficiency: simple multiplication problem accuracy (measured at the start of the first learning session) and accuracy on the initial, untimed learning block. In this untimed first complex multiplication learning block, participants could use procedural strategies to calculate the answers to the complex multiplication problems. We investigated whether these indicators of mathematical proficiency were associated with (i) learning scores on the first learning session and (ii) benefit of sleep (i.e. difference between recall scores for the sleep and wake learning conditions) for the larger sample (*n* = 70). We found that learning scores were associated with simple multiplication accuracy, *r* = 0.49, *p* < 0.001, but not scores on the untimed learning block, *r* = 0.20, *p* = 0.100. By contrast, the difference between recall scores for the sleep and wake learning conditions was associated with scores on the untimed learning block, *r* = −0.26, *p* = 0.032, but not simple multiplication accuracy, *r* = 0.18, *p* = 0.132.

## 4. Discussion

Here we found, for the first time, a sleep-related benefit in recall for learning complex multiplication facts. The effect size for this benefit (0.33–0.51) is slightly smaller than that identified in a meta-analysis of studies using similar designs for word-pairs for reduced forgetting in the sleep versus wake condition (Hedges *g* = 0.57) [3]. This extends existing evidence of the benefits of sleep from word learning to mathematical facts. Below we first compare the process of encoding of complex multiplication problems relative to the encoding of word-pairs used in previous studies. We also discuss the implications that the benefit of learning prior to sleep was found regardless of initial levels of learning of the complex multiplication problems. Some limitations of the present study are also considered.

We have found that the benefit of learning before sleep extends beyond word learning to multiplication facts. One of the differences between encoding the association of a complex multiplication problem and its product compared with word-pair associations is the greater opportunity for error due to interference effects and problem size [19]. To ensure that the stimuli learnt were novel to participants, it was necessary to use stimuli with large problem sizes, i.e. a large maximum operand. Consequently, few participants met the 60% criterion typically used in studies of the benefits of sleep versus wake on cued recall of word pairs [20]. There is little semantic information attached to the operands and the product of a complex multiplication problem. The studies using word pairs with less semantic information to link the pairs, i.e. unrelated pairs, show less accuracy and a greater number of trials to reach criterion compared with related word pairs [20,21]. In one study, sleep following learning relative to wake following learning demonstrated a greater benefit for the unrelated pairs compared with the related pairs [20], although in a smaller study the learning prior to sleep condition evidenced beneficial recall scores compared to the wake condition for both the related and unrelated word pairs [21]. Therefore, like studies with little semantic information to link the two associations, the recall of complex multiplication stimuli does benefit from learning prior to sleep relative to a period of wakefulness.

A second difference between learning multiplication facts and word-pair associations is that non-retrieval, procedural strategies (such as decomposition or repeated addition) can be used to solve multiplication problems. We investigated the potential role of these procedural strategies. Unlike a previous study that found that accuracy using non-retrieval strategies was associated with accuracy using retrieval strategies of complex multiplication problems [10], there was no association between accuracy on the untimed trials, that facilitated the use of non-retrieval strategies, and the accuracy on the learning session test. However, participants who had lower accuracy on the untimed trials experienced greater benefit of the sleep condition compared with the wake condition for recall, albeit a small association. It is possible that sleep-related benefits for learning multiplication problems could particularly benefit learners who struggle with using procedural strategies to solve problems, although further work is needed to identify what strategies are used when presented with untimed multiplication problems and allowing participants to use a pen and paper for working out problems, which was not encouraged in the present study.

The present study indicated that learning prior to sleep benefits learners at all levels of initial learning, and that prior knowledge of simple multiplication problems is not associated with the extent of the sleep-related benefit for recall. This suggests that targeting learning of multiplication problems prior to sleep could benefit learners with varying memory capacities and prior knowledge of multiplication stimuli. There is mixed previous evidence for whether participants with weaker encoding of word-pairs experience greater sleep-related benefit than individuals with higher encoding [22–24]. Further research is needed to apply these findings to a real-world educational context. Future studies could evaluate whether primary school children with heterogeneous memory skills would benefit from learning novel simple multiplication problems in the evening compared with during the day.

One limitation of this study was that participants completed this study online in unsupervised conditions, and as the data indicate, not all participants were able to follow the instructions to answer the timed multiplication problems using retrieval. However, given the longitudinal design of this study and its conduct during the COVID-19 pandemic, the online data collection was necessary to ensure a sufficient sample size. Pre-registered exclusion criteria to maximize the quality of the data (taking over 1 h 45 min to complete the learning session, completing the experiment at the incorrect time, participants reporting that their sleep on the night of the sleep learning condition was greater than 2 h shorter than usual) mitigated some of the concerns over fraudulent behaviour or low effort that could occur due to lack of supervision. The 6-s time limit for responses was identified through piloting to ensure sufficient time to type a three-digit number but insufficient time to use alternative strategies. Another study has demonstrated that the use of timed questions reduced self-reported cheating compared with other strategies [25]. The rate of non-compliance with instructions to not use strategies for 50% or more of the trials was 22%, which is slightly lower than the 24–41% of self-reported cheating in student samples [25]. An additional limitation is the self-reported sleep duration, which is overestimated compared with polysomnography-assessed sleep duration [26]. In addition, night waking was not accounted for in the calculation of sleep duration, due to poor recall for night waking. Despite this limitation, another study has also demonstrated a lack of association between sleep duration and recall performance in a sleep restriction study [27]. A further limitation is the lack of other comparative stimuli with a similar complexity of encoding with the same number of presentations to conclusively demonstrate the specificity of sleep-related benefit on the recall of complex multiplication problems. However, in this study design the learning session for the complex multiplications took approximately 1 h, so the addition of further stimuli, increasing the learning sessions to 2 h would have inordinately increased participant burden and fatigue effects. Overall, further research is needed to evaluate the effect of learning multiplication problems prior to sleep on long-term recall.

In conclusion, learning complex multiplication problems prior to sleep conferred additional benefit on recall compared with learning problems during the daytime. This effect was found across all levels of initial encoding. This effect could be evaluated in an educational context for the learning of novel simple multiplication problems.

## Data Availability

The data are available at doi:10.25392/leicester.data.22148921 [28] and doi:10.25392/leicester.data.22148909 [29] and the materials are available via Gorilla Open Materials. The experiment was pre-registered: https://aspredicted.org/3mb8d.pdf [30].
